# Prevalence of dental caries lesions in Costa Rican children under 81 months living in poverty

**DOI:** 10.1590/1807-3107bor-2026.vol40.003

**Published:** 2026-03-06

**Authors:** Sylvia GUDIÑO-FERNANDEZ, Katherine MOLINA-CHAVES, Adrian GOMEZ-FERNANDEZ

**Affiliations:** (a) University of Costa Rica, Pediatric Dentistry Master’s Program, Postgraduate Study System, San José, Costa Rica.

**Keywords:** Dental Caries, Prevalence, Socioeconomic Factors, Child

## Abstract

Dental caries remains a significant public health issue, particularly among children from low-income areas, where limited access to preventive and restorative care increases the disease burden. In Costa Rica, recent data on the prevalence of caries in children under seven years of age are lacking. This nationwide study aimed to determine the prevalence and severity of dental caries lesions in Costa Rican children aged 12–81 months living in poverty and enrolled in the Centers for Education and Nutrition and Comprehensive Child Nutrition and Care Centers (CEN-CINAI), which are national childcare and nutrition programs. A cross-sectional descriptive study was conducted with 800 children from urban and rural areas. The probability-proportional-to-size (PPS) method was used to select 40 centers, with systematic random sampling of 25 children per center. Clinical examinations were performed by eight calibrated examiners following the International Caries Detection and Assessment System (ICDAS) criteria to assess oral health conditions. The inter- and intraexaminer kappa values ranged from 0.80–0.93. The overall prevalence of caries lesions was 84.81% (ICDAS codes 1–6), with 47.19% of the participants presenting moderate-to-severe carious lesions (ICDAS codes 3–6). The highest prevalence was observed in the 37–48-month age group (91.45%). Differences in the prevalence of caries by sex were more evident in younger age groups and minimal in the older age groups. Only 28.75% of the children with caries had received restorative treatment. The 37–48-month age group presented the highest prevalence of caries, with most lesions being moderate to severe and treatment coverage remaining low.

## Introduction

Oral diseases affect approximately 3.5 billion people worldwide, ranking among the 300 most prevalent conditions impacting humanity.^1^ Among oral diseases, dental caries is particularly significant, representing a major public health concern that affects both primary and permanent dentition. The World Health Organization (WHO) reported that the most prevalent oral diseases across the life span include dental caries in primary dentition (42.71%), dental caries in permanent dentition (28.7%), severe periodontal disease (18.82%), and tooth loss (6.82%).^2^ Dental caries is a multifactorial disease influenced by biological, behavioral, psychosocial, and environmental factors.

Dental caries in primary teeth during childhood exhibit significant variations influenced by socioeconomic and geographical factors, such as living in extreme poverty in rural areas, which is associated with a higher prevalence and severity of disease.^3,4^ This issue is particularly concerning in low- and middle-income countries, where socioeconomic inequalities limit access to adequate dental care, perpetuating disparities in oral health.Additional contributing factors include the frequent consumption of sugary foods and beverages, as well as poor oral hygiene.^6^

From a biological perspective, dental caries is a noncommunicable, dynamic, and dysbiotic disease that is modulated by diet, leading to mineral loss in the hard dental tissues.^7,^Initially, caries lesions are not clinically visible; however, if intraoral buffering mechanisms fail to halt the demineralization process, caries lesions can progress through the enamel and reach the dentin, causing significant structural damage.^7^

In 2022, the WHO proposed new oral health strategies to address this reality and achieve significant improvements by 2030, urging each country to prioritize efforts in oral health promotion and prevention while addressing common risk factors for noncommunicable diseases.^9^

In Costa Rica, official data on the prevalence of dental caries in preschool children are scarce. A national survey conducted in 1999 by the Costa Rican Institute for Research and Teaching in Nutrition and Health (Instituto Costarricense de Investigación y Enseñanza en Nutrición y Salud, INCIENSA) evaluated children aged 6–8 years, reporting a prevalence of caries lesions of 75.2%.^1^The average number of primary teeth with a history of disease (decayed, missing, and filled teeth (dmft) index) was 3.32, with the following distribution: 1.84 decayed teeth (55.4%), 0.97 filled teeth (31.9%), and 0.51 missing teeth (12.7%). This study used the traditional criteria established by the WHO, which define a lesion as a visible cavity that affects dentin. This approach may underestimate the true extent of the disease by disregarding the initial stages of the caries process.^11^ In contrast, a later study conducted among infants aged 12–24 months in the metropolitan area of San José also considered noncavitated incipient lesions, reporting a prevalence of 36%.^1^More recently, research using the International Caries Detection and Assessment System (ICDAS) criteria reported a caries prevalence of 96.35% in Costa Rican children aged 2–17 years enrolled in temporary care institutions.^13^

In addition to this study from Costa Rica, studies from other low- and middle-income countries, such as Chile, Brazil, and India, have also reported a high prevalence of dental caries in preschool populations living in poverty, underscoring structural inequities in oral health and limited access to care.^4-6^ These findings emphasize the global nature of this problem and reinforce the need for cross-country comparisons and shared public health strategies aimed at mitigating early childhood caries in socioeconomically disadvantaged settings.

Understanding and documenting the prevalence and severity of dental caries in disadvantaged populations is critical not only for mitigating caries in Costa Rica but also for guiding public health actions in countries with similar socioeconomic and health care challenges.^2^

The aim of this study was to determine the prevalence and severity of dental caries lesions in a group of Costa Rican children aged 12–81 months who were living in poverty, both in urban and rural areas, using the ICDAS system. Additionally, this study aimed to assess the availability and coverage of preventive and restorative care provided to this population.

## Methods

### Study design and population

#### Sampling framework

This cross-sectional descriptive study was conducted from March–June 2013. The sample included children younger than seven years of age who were enrolled in the Centers for Education and Nutrition and Comprehensive Child Nutrition and Care Centers (Centros de Educación y Nutrición y de Centros Infantiles de Nutrición y Atención Integral, CEN-CINAI) programs, operated by the Ministry of Health of Costa Rica. These centers serve children from families facing extreme poverty, severe or moderate malnutrition, and social vulnerability, including children of adolescent mothers and children of pregnant women living in poverty.^14,1^

The sampling framework was based on the selection of centers for data collection, using the official CEN-CINAI establishment list, which includes information on regions, provinces, cantons, districts, and age groups (0–2 years, 2–7 years, and 7–13 years of age), as well as care networks. The National Directorate of CEN-CINAI and the Department of Dentistry of the Ministry of Health of Costa Rica provided the necessary authorizations, and population lists of registered children younger than seven years of age enrolled at each center.

A two-stage stratified sampling method was applied to ensure a representative sample of approximately 10%, using a procedure known as probability proportional to size (PPS), where size refers to the number of enrolled children within the target age group. Only centers with a daily attendance of 15 or more children were included. Among the 461 establishments, 390 met the selection criteria.

Key considerations included the time available for data collection, the financial and human resources required for the study, and travel plans to reach CEN-CINAI centers outside the central region. Additionally, a maximum capacity of 50 children per day was established to conduct clinical examinations.

**Figure 1:**
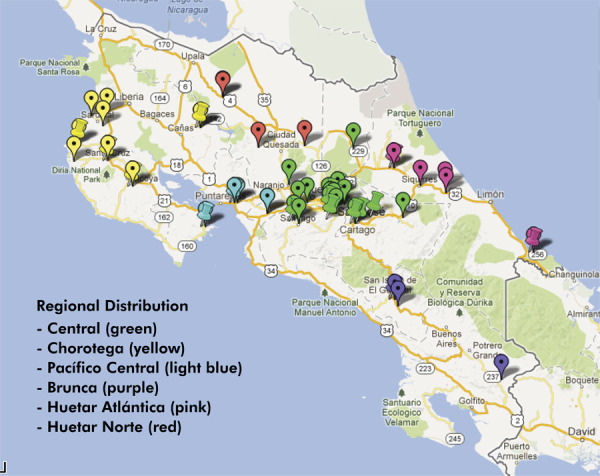
CEN-CINAI Centers included in the study and their geographic distribution

In the second stage, a sample of 40 CEN-CINAI centers was selected; within each center, a systematic subsample of 25 children was drawn, ensuring an equal probability of inclusion for all children within the target age range. Across the six socioeconomic regions defined by the Ministry of National Planning and Economic Policy (Ministerio de Planificación Nacional y Política Económica, MIDEPLAN), 42.34% of the centers were from the Central Region (17 centers), whereas the remaining 57.66% were distributed across other regions: the Chorotega Region (8 centers), Central Pacific Region (4 centers), Brunca Region (3 centers), Huetar Atlantic Region (4 centers), and Huetar North Region (4 centers) (Figure 1).

#### Subsampling of children

According to the information provided by the administration of each CEN-CINAI center, the following procedure was followed for participant selection:

- On the basis of the official list, the total number of enrolled children aged 12–81 months was determined.
- If the number of enrolled children did not exceed 25, all the children were included. If the number was higher than 25, the sampling interval (k) was calculated as k=n/25. For example, if 55 children were enrolled, the interval was calculated as k=55/25=2.2.
- A random number between 1 and 2 was selected, and this number was increased by 2 until 25 children were included in the sample. If a selected child was absent, the next child in the sequence was chosen.

A total of 803 children were selected and examined (393 boys and 410 girls). However, 3 children under 12 months of age were excluded from the final analysis to maintain consistency with the study’s eligibility criteria, resulting in an analytical sample of 800 children.

#### Clinical examination

Examinations were conducted onsite at CEN-CINAI facilities in spacious, well-ventilated environments, using portable dental units. Each unit was equipped with individual lamps and air compressors. Each examination team consisted of an examiner, a recorder, and an assistant, who performed preexamination tooth brushing for each participant. Each examiner used a headlamp, a standard no. 5 dental mirror, and, when diagnostic uncertainty arose, a WHO 11.5 spherical periodontal probe to avoid enamel damage during tactile inspection.

The evaluation team included eight calibrated examiners who were trained by ICDAS international gold standard experts. The inter- and intraexaminer kappa coefficients ranged from 0.80–0.93, ensuring diagnostic consistency. Examiner calibration was verified through a pilot study with 30 children (who were not included in the final study sample), and periodic reinforcement sessions were conducted throughout the main study to ensure consistency and accuracy in the detection of caries lesions.

The clinical data recording instrument was a precoded form designed for primary teeth. The teeth were examined first in their natural moisture state, followed by examination after gentle air drying using a triple syringe, and/or sterile gauze.

The ICDAS was used for the diagnosis of dental caries. The ICDAS system classifies caries lesions on a scale from 0 to 6, where 0 indicates a tooth surface with no evidence of carious lesions; 1 and 2 represent initial enamel changes, detected by air drying (code 1) or visible on a wet surface (2); 3 and 4 correspond to localized enamel breakdown without dentin exposure (3) and a shadow of dentin beneath an intact surface (4); and 5 and 6 indicate cavitated lesions with visible dentin affecting up to half (5) or more than half (6) of the tooth surface.^16^

This system allows for the identification of caries lesions, from initial enamel demineralization to advanced cavitated lesions affecting dentin. The use of the ICDAS provides a more sensitive and standardized method than traditional indices such as the dmft index, facilitating early diagnosis and enabling minimal intervention strategies. The validity of the ICDAS system has been supported by histological correlation studies, which have demonstrated the ability of the system to discriminate lesion severity and activity.^16^

## Statistical analysis

The statistical analysis was performed using the Statistical Package for the Social Sciences (SPSS for Windows, version 22.0, IBM Inc., Armonk, USA). Descriptive statistics were applied to analyze the data and categorize the results by age and sex, including the calculation of absolute and relative frequencies and cumulative percentages. The chi-square test was used to compare categorical variables such as the prevalence and distribution of caries across age and sex groups.

We calculated the prevalence of one or more carious lesions per age group to determine the caries frequency. Additionally, the mean number of carious lesions per tooth was calculated as an indicator of disease severity, with the standard deviation (SD) used to assess variability.

Proportion comparison tests were performed to compare the prevalence of caries among different age groups. For comparisons of mean caries and restoration counts among groups, one-way analysis of variance (ANOVA) was applied when normality assumptions were met; otherwise, the Kruskal-Wallis test was used. When comparing only two groups, Student’s t-test or Mann-Whitney U test was applied depending on the data distribution.

The proportion of carious lesions relative to the total number of examined teeth was calculated to evaluate the burden of disease in primary dentition. Progression trends were determined by comparing the prevalence of caries across age groups.

Finally, the coverage of restorative treatments was assessed by calculating the percentage of restored teeth relative to carious teeth. The relationship between caries and restored teeth was further explored using correlation analysis, and, if applicable, logistic regression to determine associations between caries severity and the amount of restorative care received.

## Ethical considerations

The study was approved by the Institutional Committee of the University of Costa Rica (VI-3058-2012), and all parents of the participants signed an informed consent form before the study began. Informed consent was obtained for 100% of the children, and the parents or guardians were thoroughly informed about the study objectives and procedures, ensuring compliance with the ethical principles of autonomy, beneficence, and nonmaleficence.

The children’s parents or guardians received detailed explanations regarding the study and signed an informed consent form at all stages of data collection. The study was conducted under the highest ethical standards, ensuring the protection of the rights and well-being of all participants at all times.

## Results

### Age and sex distribution

The study sample initially consisted of 803 children. However, in alignment with the inclusion criteria, 3 children under 12 months of age were excluded from the analysis, resulting in a final analytical sample of 800 children (392 boys and 408 girls; ratio 0.96:1). The largest age groups were those aged 49–60 months (34.13%) and 37–48 months (29.25%), accounting for 63.38% of the total sample. The smallest age groups were those aged 13–24 months (3.13%) and 73+ months (2.50%) (Table 1).

**Table 1:** Age and sex distributions of Costa Rican preschool children.

Age group (months)	Female	Male	Total	Percent (%)	Male-to-female ratio
13–24	8	17	25	3.13	2.1
25–36	42	47	89	11.13	1.1
37–48	125	109	234	29.25	0.9
49–60	136	138	274	34.25	1.0
61–72	86	72	158	19.75	0.8
73+	11	9	20	2.50	0.8
Total	408	392	800	100	1.0

### Prevalence of dental caries by age and sex

Overall, 84.81% of the children (n = 681; 95%CI: 81.2–87.9) presented at least one caries lesion (ICDAS codes 1–6). Among these children, 44.34% presented with noncavitated incipient lesions (ICDAS codes 1–2), and 47.19% had moderate-to-severe lesions (ICDAS codes 3–6). Only 15.19% of the children were caries-free (ICDAS code 0) (Table 2).

**Table 2:** Prevalence by age group and severity of caries.

Age group (months)	Total individuals	Individuals with caries	Prevalence (%)	No Caries	Mild caries (ICDAS codes 1-2)	Moderate-severe caries (ICDAS codes 3-6)
13–24	25	8	32.0	17	6	2
25–36	89	63	70.79	26	41	22
37–48	234	214	91.45	20	96	118
49–60	274	235	85.77	39	99	136
61–72	158	142	89.87	16	54	88
73+	20	19	95.0	1	6	13

The prevalence of caries increased progressively with age. The lowest prevalence was observed in the 13–24 month age group (32%), whereas the highest prevalence was observed in the 73+-months age group (95%). A notable increase was observed between the 13–24-month (32%) and 25–36-month age groups (70.79%), suggesting early caries onset. After the age of three years, the prevalence remained consistently high, above 85%.

With respect to sex distribution, only minor descriptive differences were observed across age groups. In the 13–24-month age group, boys had a greater caries prevalence than girls did, whereas in the 25–36-month age group, the opposite trend was observed. In the 73+-month age group, 100% of the girls had caries, whereas 88.9% of boys did. These differences were descriptive only; no statistical tests were conducted to assess significance.

#### Severity of caries and ICDAS code distribution

Among all the children examined, the most frequently recorded ICDAS code was 2 (36.50%), which corresponds to distinct visual changes in the enamel without cavitation. This was followed by code 6 (22.13%) and code 5 (14.63%), indicating that a considerable proportion of advanced cavitated lesions involved dentin. Less frequent codes included codes 1 (1.25%), 4 (4.25%), and 3 (6.38%), suggesting a relatively lower prevalence of early-stage enamel lesions, underlying dentin shadows, and localized enamel breakdown.

As shown in Table 3, 14.88% of the children were caries free (ICDAS code 0), whereas 85.13% presented with at least one lesion. More severe lesions (ICDAS codes 3–6) appeared to be more common in older children, whereas early-stage lesions (ICDAS codes 1–2) were more common in the younger children. This pattern reflects the natural progression of caries severity with age in this population.

**Table 3:** Distribution of ICDAS caries codes and proportion of cases in preschool Costa Rican children.

Caries code	Number of children	Percentage (%)	Cumulative percentage (%)	Proportion of cases with caries (%)
0	119	14.88	14.88	-
1	10	1.25	16.13	1.47
2	292	36.50	52.63	42.88
3	51	6.38	59.0	7.49
4	34	4.25	63.25	4.99
5	117	14.63	77.88	17.18
6	177	22.13	100.0	25.99

ICDAS: International Caries Detection and Assessment System. Codes represent the following clinical conditions: Code 0: Sound tooth surface (no evidence of caries; may include developmental defects such as enamel hypoplasia, hypomineralization, or wear); Code 1: First visual change in enamel (seen only after drying); Code 2: Distinct visual change in enamel (seen without drying); Code 3: Localized enamel breakdown without visible dentin; Code 4: Underlying dark shadow from dentin; Code 5: Distinct cavity with visible dentin; Code 6: Extensive distinct cavity with visible dentin. The column “Proportion of Caries Cases (%)” refers to the percentage of children with carious lesions (n = 681) who presented each ICDAS code.

#### Restorative treatment coverage

Despite the high prevalence of dental caries, restorative treatment coverage remained disproportionately low across all age groups. For example, in the 25–36-month age group, 70.79% of the children had carious lesions, yet only 12.36% received restorative treatment. The lowest coverage rate was observed in the 13–24-month age group (8%), whereas the highest coverage rate was observed in the 73+-month age group (65%).

Across the total sample, only 28.64% of the children with caries received restorative treatment, highlighting a significant gap between disease burden and access to curative care (Table 4).

**Table 4:** Restorative treatment coverage by age group.

Age group (months)	Total children	Children with caries	Children with restorations	Caries-to-restorations ratio
		n (%)	n (%)	
13–24	25	8 (32.0)	2 (8.0)	4
25–36	89	63 (70.79)	11 (12.36)	5,7
37–48	234	214 (91.45)	52 (22.22)	4,1
49–60	274	235 (85.77)	86 (31.39)	2,7
61–72	158	142 (89.87)	66 (41.77)	2,2
73+	20	19 (95.0)	13 (65.0)	1,5
Total	800	681 (84.81)	230 (28.75)	3

**Figure 2:**
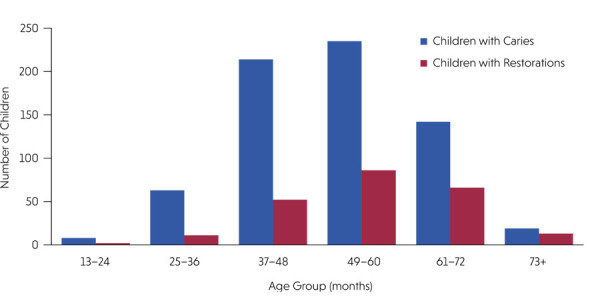
Comparison of the number of children with carious lesions and those who received restorative treatment by age group

Figure 2 illustrates the treatment gap observed across all age groups in Costa Rican preschool children aged 12–81 months, comparing the number of children with caries and those who received restorative treatment by age group. This visual representation highlights the disparity between the burden of disease and access to curative care.

## Discussion

According to a recent WHO report, the global prevalence of dental caries lesions in primary dentition is 43%, affecting 69% of WHO member countries.^2^ Untreated dental caries lesions are widely recognized to lead to serious consequences, including an increased risk of lesions in future permanent teeth.^3^ Our data reflect trends similar to those observed in low- and middle-income countries, where socioeconomic conditions profoundly influence both the prevalence and severity of caries lesions.^17^

This study, in which primary teeth were exclusively analyzed according to the age range of the participants, revealed a substantially higher caries prevalence than that reported by the WHO, reaching 84.81%, with 47.19% of caries lesions classified as moderate-to-severe lesions (ICDAS codes 3–6). These differences in prevalence rates may be partially explained by the methodology used for clinical examination and data collection. In Costa Rica, the ICDAS visual-tactile method allows for the detection of all carious lesions, from noncavitated to severe stages. In contrast, the WHO reports typically use the dmft/DMFT index, where recorded lesions correspond exclusively to cavitated lesions with evident dentin decay. The use of the ICDAS provided a more sensitive and detailed profile of caries progression in early childhood, which would not have been captured by conventional indices.

When our findings are compared with those of previous studies, it is evident that the prevalence of dental caries in children under four years of age varies significantly depending on region and socioeconomic conditions. For example, a study conducted in Yucatán, Mexico, reported a 35% prevalence of dental caries in children aged 9–48 months, using the dmft index (referred to as ceo-d in Spanish-language studies) in combination with the Pitts and Fyffe scale to classify early-stage lesions.^18^

Unlike the ICDAS method used in our study, the traditional dmft index cannot be used to evaluate noncavitated incipient lesions; however, the additional scale employed in their study allowed for the identification of early carious lesions in 73% of caries cases.

In our study, the prevalence of caries was reported as the proportion of affected individuals by age group, without the application of statistical tests to evaluate associations between variables. Therefore, comparisons of these results with those of analytical studies should be performed with caution. Nevertheless, our data revealed noticeable variations in the prevalence of caries between boys and girls, particularly in the 13–24-months age group, where boys presented a greater incidence of carious lesions. These descriptive differences may differ from those reported in analytical studies, such as a study conducted in Mexico, which reported no significant sex differences in the incidence of early childhood caries.^18^

A study conducted in Santiago, Chile, reported a prevalence of early childhood caries of 63% among children aged 24 to 71 months living in socially vulnerable conditions, as assessed using the dmft index. That study also identified the maternal education level as a key determinant, indicating that children whose mothers had lower educational levels were more likely to develop caries.^19^ These findings support our observation that social conditions play a crucial role in oral health outcomes.

Moreover, in Andes, Colombia, a study of preschool children using the ICDAS system reported the highest caries prevalence among 3- and 4-year-olds, with 88.4% of the children presenting with caries when initial and moderate lesions were considered and 38.2% presenting with caries when only severe cavitated lesions were included.^20^ The severity of caries lesions increased progressively with age, reaching 53.6% in 5-year-old children and 100% when all lesion stages were included. These findings are consistent with those of our study, where caries severity also increased significantly with age, especially in the 37–48-month and 49–60-month age groups.

A previous Costa Rican study analyzing caries lesions in 414 children aged 12 to 24 months, which also considered the presence of noncavitated incipient lesions, reported a 36% prevalence.^12^ This value closely matches the value of 32% observed in our study for the same age group, with only a slight decrease. Both studies highlight the limited restorative coverage for this population, as children with affected teeth had not received treatment. Additionally, that report emphasized the impact of feeding practices, such as frequent consumption of fermentable carbohydrates and prolonged nocturnal breastfeeding without oral hygiene habits.

Our results indicate that both caries prevalence and lesion severity increase with age, especially after 24 months. This pattern has been documented in other descriptive studies, where the increase in caries prevalence is associated with the early introduction of cariogenic diets and poor oral hygiene.^21^

A multicenter study conducted in ten Latin American countries, including Costa Rica, revealed that sugary beverages were introduced at an average age of 7.7 months in Costa Rica, which is earlier than the regional average. Additionally, 28% of the children consumed sugary products four or more times per day.^22^ This early and frequent exposure increases the risk for early childhood caries.

Access to healthcare is also crucial in addressing inequalities. For example, a study conducted in Appalachia reported sex-based differences in caries experience, with boys showing a greater prevalence of caries in primary dentition.^23^ In Costa Rica, however, our findings revealed minimal sex differences in older age groups, possibly reflecting a more equitable healthcare system.

A key finding of this study is the considerable gap between caries prevalence and restorative treatment coverage. This treatment gap has also been observed in other low-income populations, including in a Spanish study that reported that the high prevalence of caries in young children was not matched by adequate restorative care.^24^ Although restoration rates increased with age in our sample, they remained disproportionately low across all age groups, underscoring a consistent disparity between disease burden and treatment coverage, as seen in our study and in previous studies on vulnerable populations.

Experiences from countries such as Brazil and Sweden support the implementation of preventive strategies and public health policies. Programs such as Brasil Sorridente have demonstrated success by integrating oral health into the public health system,^25^ whereas Sweden’s universal dental care system prioritizes early intervention.^26^

In Costa Rica, the National Oral Health Plan 2022–2032 recognizes children as a priority population.^27^ However, ensuring effective implementation and monitoring is essential, especially in vulnerable groups served by childcare and nutrition programs such as CEN-CINAI.

These efforts should be complemented by targeted educational and preventive interventions for parents/caregivers, children, and teachers, which are tailored to the cultural and socioeconomic context of each community. This is especially relevant during the preschool years in childcare and nutrition centers for disadvantaged children, such as CEN-CINAI, where early caries risk interception is most effective and where these programs can serve as ideal platforms for early intervention within a comprehensive national oral health strategy.

This study has several limitations. First, it was not possible to collect or include detailed sociodemographic information such as parental education or household income levels, which could have provided further insight into risk factors for dental caries. However, information on the geographic region of each participant was obtained and will be analyzed in a separate publication focused on regional comparisons. Second, the cross-sectional design of the study limits the ability to infer causal relationships between the identified variables. Third, absenteeism on the day of clinical examination and logistical constraints that prevented the inclusion of all eligible children may have introduced selection bias. Despite these limitations, the study’s large, nationwide sample and the use of a validated diagnostic system (ICDAS) increase the robustness and relevance of the study’s findings.

## Conclusions

This study highlights the significant burden of dental caries among Costa Rican preschool children living in poverty, particularly as the increasing severity of lesions with age. Younger children were more likely to be caries free, whereas older children presented with more advanced lesions.

Restorative treatment coverage was low across all age groups, indicating a considerable gap between oral health needs and access to care.

These findings provide updated national data based on standardized diagnostic criteria and underscore the importance of early detection and comprehensive surveillance. In vulnerable populations, the use of sensitive diagnostic tools such as the ICDAS is essential for guiding timely and effective public health interventions.

## Data Availability

The datasets generated during and/or analyzed during the current study are available from the corresponding author on reasonable request.
